# Talk, trust and time: a longitudinal study evaluating knowledge translation and exchange processes for research on violence against women

**DOI:** 10.1186/1748-5908-6-102

**Published:** 2011-09-06

**Authors:** C Nadine Wathen, Shannon L Sibbald, Susan M Jack, Harriet L MacMillan

**Affiliations:** 1 Faculty of Information and Media Studies, The University of Western Ontario, London ON Canada; 2 Faculty of Health Sciences, The University of Western Ontario, London ON Canada; 3 School of Nursing, McMaster University, Hamilton, Ontario, Canada; 4 Departments of Psychiatry and Behavioural Neurosciences, and of Pediatrics, Offord Centre for Child Studies, McMaster University, Hamilton, Ontario, Canada

## Abstract

**Background:**

Violence against women (VAW) is a major public health problem. Translation of VAW research to policy and practice is an area that remains understudied, but provides the opportunity to examine knowledge translation and exchange (KTE) processes in a complex, multi-stakeholder context. In a series of studies including two randomized trials, the McMaster University VAW Research Program studied one key research gap: evidence about the effectiveness of screening women for exposure to intimate partner violence. This project developed and evaluated KTE strategies to share research findings with policymakers, health and community service providers, and women's advocates.

**Methods:**

A longitudinal cross-sectional design, applying concurrent mixed data collection methods (surveys, interviews, and focus groups), was used to evaluate the utility of specific KTE strategies, including a series of workshops and a day-long Family Violence Knowledge Exchange Forum, on research sharing, uptake, and use.

**Results:**

Participants valued the opportunity to meet with researchers, provide feedback on key messages, and make personal connections with other stakeholders. A number of factors specific to the knowledge itself, stakeholders' contexts, and the nature of the knowledge gap being addressed influenced the uptake, sharing, and use of the research. The types of knowledge use changed across time, and were specifically related to both the types of decisions being made, and to stage of decision making; most reported use was conceptual or symbolic, with few examples of instrumental use. Participants did report actively sharing the research findings with their own networks. Further examination of these second-order knowledge-sharing processes is required, including development of appropriate methods and measures for its assessment. Some participants reported that they would not use the research evidence in their decision making when it contradicted professional experiences, while others used it to support apparently contradictory positions. The online wiki-based 'community of interest' requested by participants was not used.

**Conclusions:**

Mobilizing knowledge in the area of VAW practice and policy is complex and resource-intensive, and must acknowledge and respect the values of identified knowledge users, while balancing the objectivity of the research and researchers. This paper provides important lessons learned about these processes, including attending to the potential unintended consequences of knowledge sharing.

## Background

Data on the prevalence [1-3], consequences [4-7], and costs [8,9] of intimate partner violence (IPV) against women attest to its persistent and devastating impact on the lives of women, their children, and society. It has been almost 20 years since IPV was declared to be a major public health problem [10], yet many gaps remain regarding effective approaches to detecting and responding to it [11-14], which have led to debates and conflicting advice to health and social service providers and policy decision makers [15].

### McMaster Violence Against Women (VAW) Research Program and Knowledge Translation and Exchange (KTE) Project

**Figure 1:**
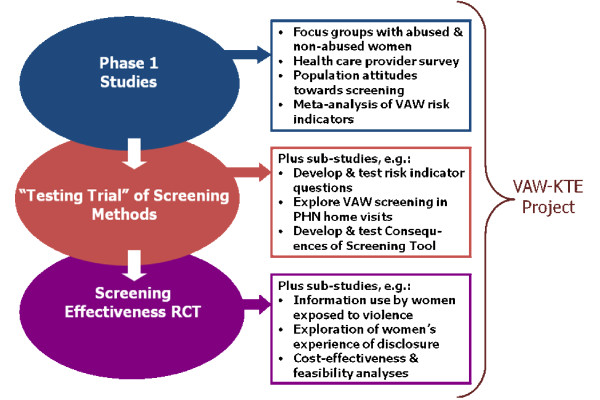
**McMaster VAW Research Program**. A schematic of the research program and projects from with the research evidence for the KTE project was drawn. VAW: violence against women; RCT: randomized controlled trial; PHN: public health nurse; KTE: knowledge translation and exchange.

In 2003, the Ontario Women's Health Council (OWHC), an advisory body to the Ontario Minister of Health and Long-Term Care, funded the VAW Research Program at McMaster University. The research program had as its primary goal answering the question: does routine screening for intimate partner violence against women presenting to healthcare settings reduce violence and improve life quality for women? The program was conducted in three phases (Figure 1), with multiple qualitative, quantitative, and mixed-methods projects designed to answer specific questions that required evidence in order to develop the main study, a randomized controlled trial (RCT) of the effectiveness of screening including 18 months of follow-up. In 2006, a group of researchers from the VAW Research Program, in partnership with policy analysts from the OWHC, were funded to begin to identify and develop the main messages arising from the completed and ongoing projects. In 2008, we received new funding for additional KTE activities focussed on the results of the screening effectiveness trial that were published in 2009 [16].

### Approach to KTE

The KTE activities described in this study were guided by the interaction model of knowledge translation [17,18], and assumed that effective KTE would require initiating and assessing 'various disorderly interactions occurring between researchers and users' [18] and understanding that researchers and knowledge users (broadly defined) are 'two communities' [18], or in the case of our identified stakeholder groups, multiple communities. The McMaster VAW Research Program utilized an integrated knowledge translation approach [19] with knowledge users representing clinical practice, community service, and public policy decision-making constituencies involved from the outset as members of the research team. These partners, in addition to helping to shape the design of the research studies, were key resources when planning and implementing the KTE strategies described below.

The interaction model also stresses the development and evolution of 'relationships between researchers and users at different stages of knowledge production, dissemination and utilization' [18], and assumes that more numerous and intensive interactions between researchers and users will lead to greater potential for use of the knowledge; this rationale underpinned our approach to multiple contacts (frequency and type) over time, both with organizations and individuals. Further, the factors that mediate knowledge utilization include, according to Oh and Rich [17]: characteristics of the information, organizational characteristics, motivations and attitudes of the knowledge users, and the nature of the knowledge gap/problem to be addressed. The contextualization of research messages and KTE strategies to take into account the second and fourth factors above was a key priority in our KTE processes [20-22].

Finally, to map our findings across key stages of KTE processes as generally articulated in the literature [21,23], our questions were asked, and results are presented, according to the following: dissemination and uptake; sharing and use; and impact. Within the 'sharing and use' domain, recognizing that 'knowledge utilization' is a multi-faceted phenomenon, we frame the concept in three ways consistent with the KTE literature [18,24,25]: conceptual/enlightening use (*i.e*., 'to provide better understanding or insight about an issue'); symbolic/selective use (*i.e*., 'to support or refute an existing belief, policy, process, or course or action'); or instrumental/direct use (*i.e*., 'to propose a new policy, process or course of action').

The overall goal of our KTE project, therefore, was to ensure that results arising from the research were identified early, developed appropriately, and shared with key audiences, including policy decision makers, healthcare practitioners, community service providers, and advocates. In this paper, we report on the four-year study that identified and documented how stakeholders received, engaged with, and used (or not) the research knowledge shared with them via a series of KTE strategies. Our specific research questions were: How do recipients of research evidence perceive the utility of specific KTE strategies in the area of violence against women? What factors, according to those receiving research evidence, influence the uptake, sharing, and use of the new knowledge? And what kinds of use are made of research findings? We also reflect on the 'lessons learned' from this longitudinal project that might be applicable to KTE efforts and evaluation more broadly.

## Methods

### Design

A longitudinal cross-sectional design, applying concurrent mixed data collection methods [26,27], was used to describe and assess our KTE processes and their impact on the types of knowledge utilization described above. Phase 1 of the study examined the process for developing initial research messages and sharing them with stakeholders at an interim point in the research program. Phase 2 focused on uptake and use of the final results of the screening trial. Table 1 provides an overview of the KTE activities conducted from 2006 to 2009, and the data collection methods (to April 2010) used to evaluate their impact.

**Table 1:** Overview of knowledge translation and exchange (KTE) activities and evaluation strategies

KTE Activity	Description and Participants	Evaluation Approach
***Phase 1 (2006 and 2007)***		

Key message development (VAW Research Phase 1 Studies and Testing Trial) (Spring and Summer 2006)	Core research team and policy partner/funder drafted key messages; the wider VAW research team reviewed them, and they were formatted for stakeholder audiences.	Observation and journaling by core research team re: process

Stakeholder workshops (October 2006)	82 stakeholders attended one of three half-day workshops in Toronto, Ottawa, or London Ontario.	Workshop evaluation survey (Fall 2006) (n = 75) In-depth telephone interviews (Winter 2006/7) (n = 20) Follow-up online survey (Winter 2007) (n = 33)

Online community of interest (launched Spring 2007)	In response to request from stakeholders, created an online wiki-based site to continue interaction.	Usage data

***Phase 2 (2008 and 2009)***		

Key message development (Screening Trial) (late 2008 to early 2009)	Core research team and policy partners/funders drafted key messages; wider VAW research team reviewed them, and they were formatted for stakeholder audiences, including media talking points.	Observation and journaling by core research team re: process

Family Violence Knowledge Exchange Forum (January 2009)	Day-long meeting, in Toronto, of 76 stakeholders and 11^1 ^researchers from the McMaster VAW Research Program. Focus on high-level key messages and discussion of policy and practice implications.	Forum evaluation survey (n = 38) Analysis of Forum small group transcripts (n = 10 groups) Participant follow-up survey (Summer 2009) (n = 21) Follow-up interviews (Fall 2009 to Winter 2010) (n = 12)

Media (Summer 2009)	Publication of screening trial in JAMA in August 2009 led to significant media interest	Included questions about media exposure in follow-up interviews

^1^Three members of the research team who attended were knowledge user partners with clinical leadership roles; VAW: violence against women; JAMA: Journal of the American Medical Association

### Phase 1: 2006 and 2007

#### Key message development

The team reviewed VAW research program reports, including results from eleven projects (Figure 1, Phase 1 and 'Testing Trial' [28]), to identify relevant findings. Key messages were identified using a structured, iterative process, including input from the research team and key policy partners/funders, and presented using appropriate formats [29] (see Additional File 1).

#### Stakeholder workshops and evaluation

In October 2006, we held half-day workshops in London, Toronto, and Ottawa, Ontario attended by 82 stakeholders. Each began with a networking lunch, followed by research project presentations, key messages, and preliminary synthesis, with time for discussion. Participants were then divided into groups, facilitated by a research team member, and discussed two questions: 'what are the implications of these findings?' and 'what should happen next?' followed by reporting-back and plenary discussion.

Primary evaluation methods were: an evaluation survey immediately post-workshop (n = 75); an online follow-up survey about three months post-workshop (n = 33); and in-depth telephone interviews about six months post-workshop with participants who had consented to follow-up (n = 20). The evaluation survey consisted of 10 structured questions asking about work setting, role and decision-making responsibilities, level of previous involvement with our research, and experiences during the workshop, including overall usefulness.

The online follow-up survey asked similar questions to those above, and questions regarding influence/impact of using the research, as well as ongoing interaction between stakeholders and the research team.

The follow-up telephone interviews used a descriptive qualitative approach [30] to further probe the evaluation survey results and to explore the impact that the workshops had on subsequent decision making. We purposefully sampled from the three workshop sites at least two stakeholders from each of the following groups: public policy, healthcare providers (hospital and community-based), social service providers, and women's advocates. The semi-structured interview guide asked about their experience of the workshop, whether they had shared or used the research (and if so, how and to what effect), or planned to do so. Interviews lasted about an hour and were audio-recorded (with permission).

#### Development of an online community of interest

Participants at all three workshops endorsed the idea of an interactive website, using 'Web 2.0' technologies, to allow ongoing interaction; we therefore developed the 'online community of interest' (http://www.VAWCommunity.ca; link no longer active). Launched in March 2007, and using a wiki platform, the site included static documents and information (*e.g*., summaries, meeting notes and slides, *et al*.) and interactive areas, where users were invited to edit meeting notes to reflect their memory of the discussions, and edit key messages to make them more relevant or user-friendly. The online follow-up survey was linked to the site.

### Phase 2: 2008 and 2009

#### Key message development

As with Phase 1, an initial step was to develop key messages from the screening trial in the context of both our previous messages and the broader evidence-base (Additional file 1). A particular challenge was the nature of the main results of the trial: for one primary outcome, recurrence of violence, the difference was not statistically significant; for the second, quality of life, there was a small clinically non-significant difference that also became statistically non-significant following multiple imputation to account for data loss. The differences between all secondary outcomes were not statistically significant, with the exception of depressive symptoms, which showed the same pattern as quality of life. To help enhance the relevance and clarity of the results, we held meetings with key Ontario policy stakeholders (see details in Additional file 2); we used their input to develop the final key messages.

#### Family violence knowledge exchange forum

In January 2009, we hosted the Family Violence Knowledge Exchange Forum in Toronto, Ontario. This included, in addition to members of the research team, policy makers (federal and provincial), health and social service leaders, women's and children's advocates, and other family violence researchers from across Canada. This day-long interactive event featured brief research presentations (oral and poster), plenary discussions, and 10 small group discussions that followed presentation of key messages [31]. Given feedback from the Phase 1 workshops, we minimized presentation of data, and maximized time for discussion; key messages were presented as 'actionable' [32] messages in 10 minutes with minimal data, graphs, or research jargon.

#### Forum evaluation

In addition to audio- and video recording the session, field notes and post-meeting debriefing, we used the following methods to evaluate the forum:

#### Small group discussions (n = 10 groups)

To understand the initial impact of the screening trial results, we captured stakeholders' reactions to the key messages by asking them to consider and discuss them immediately after they were presented (see discussion questions in Additional file 2). The discussion at each table was audio-recorded (those who did not want their comments recorded could pause the recorder while speaking). Based on feedback from the Phase 1 workshops, there was no formal group moderation; rather, the research team circulated to answer questions regarding the research. Table seating of 8 to 10 participants was pre-assigned to mix groups by sector, role, and geography.

#### Evaluation survey (n = 38)

Attendees were asked to complete an exit survey that used the same questions and format as the Phase 1 workshop evaluation.

#### Follow-up survey (n = 21)

Approximately six months after the forum, stakeholders who gave permission for follow-up were sent an email invitation to complete an online survey. The survey had 18 structured questions similar in content to those described above.

#### Follow-up interviews (n = 12)

Qualitative semi-structured interviews using the same methods described above for the Phase 1 follow-up interviews, and probing the same kinds of questions as the Phase 2 follow-up survey, were conducted 9 to 12 months following the forum. Additional KTE activities not directly assessed in this project are described in Additional File 2.

### Data Analysis

Data from post-meeting and follow-up evaluation surveys were entered into Excel and/or SPSS, cleaned and checked by a research assistant, and descriptive statistics generated. Transcript data from meeting small groups and follow-up interviews were transcribed verbatim, cleaned, organized in NVivo^©^, and analysed using directed content analysis [33], with each coder using a list of predetermined codes based on the concepts explored in the interview or group. Codes were then collapsed into primary categories. To ensure trustworthiness of the data, each transcript was independently reviewed, and key themes identified, by two study investigators, with review of synthesized results by additional collaborators who had attended the workshops and forum. Concurrent triangulation of the results [27] within each phase allowed us to integrate the qualitative and quantitative data for more complete interpretation of participants' experiences and perspectives, as well as using emerging findings from Phase 1 to inform the development of the KTE strategies and evaluation methods used in Phase 2.

### Ethical considerations

Phase 1 of the study was reviewed and provided a waiver (*i.e*., deemed to pose no potential risk to consenting participants) by the McMaster University Faculty of Health Sciences- Hamilton Health Sciences Research Ethics Board (REB). Phase 2 was approved by the University of Western Ontario Non-Medical REB (protocol #15789S).

## Results

### Participant Characteristics

Given the nature of the data collection methods and ethical requirements regarding participant anonymity, each data collection point represents a separate sample--*i.e*., this is not a cohort of individuals followed across time, but rather individuals who self-selected participation at these various points in the study; 190 stakeholders were invited to the 2006 workshops and 82 attended; 217 were invited to the forum, and 76 attended; 139 stakeholders (34%) were invited to both events, and 15 (8.9%) attended both. Thus, while there was minimal overlap between the samples of respondents to our data collection approaches, there was certainly growing awareness of the work among the overall targeted group of stakeholders (individuals and organizations) who received invitations and interacted with the research office re: RSVPs and other meeting logistics. Tables 2 and 3 describe the types of workplaces (Table 2) and decision-making roles (Table 3) reported by respondents in Phases 1 and 2. A wide range of settings and roles were represented, with 56% of participants reporting having multiple decision-making roles, and a significant number reporting an overlap between clinical/service delivery and planning/administrative roles. Additional file 2 provides an overview of the samples participating at each stage of data collection, and specific sub-sample sizes are specified in the Tables.

**Table 2:** Workplace types reported by participants (Phase 1 workshop evaluation and online follow-up survey; Phase 2 forum evaluation)

Workplace type^1^	Phase 1: Workshop Evaluation (n = 75) % (n)	Phase 1: Follow-Up Survey (n = 33) % (n)	Phase 2: Forum Evaluation (n = 38) % (n)
Community based service organization (*e.g*., Shelter)	24% (18)	9% (3)	8% (3)

Advocacy group	9% (7)	0% (0)	3% (1)

Acute or primary healthcare service organization	32% (24)	30% (10)	13% (5)

Public health unit or agency	23% (17)	21% (7)	13% (5)

Government department (provincial, federal, municipal)	17.3% (13)	24% (8)	16% (6)

University department and/or research centre	7% (5)	0% (0)	34% (13)

Other (write-in)	5% (4)^2^	15% (5)^3^	13% (5)^4^

^1^respondents could indicate more than one type; ^2 ^legal, police, professional association, grassroots; ^3^university student, professional association, municipal government, police, information centre; ^4 ^minister, lawyer, regulatory body, provincially funded support and funding agencies, police

**Table 3:** Types of decision-making roles (Phase 1 workshop evaluation and online follow-up survey; Phase 2 forum evaluation and online follow-up survey)

Decision-making role	Phase 1: Workshop Evaluation^1^ (n = 74, 1 missing) % (n)	Phase 1: Follow-Up Survey^2^ (n = 33) % (n)	Phase 2: Forum Evaluation^1^ (n = 35, 3 missing) % (n)	Phase 2: Follow-Up Survey^2^ (n = 16, 5 missing) % (n)
Clinical care/service delivery decisions	41% (30)	12% (4)	23% (8)	6.3%(1)

Planning/programming decisions	51% (38)	18% (6)	31% (11)	31.3%(5)

Administrative decisions	41% (30)	27% (9)	9% (3)	0% (0)

Public policy decisions	19% (14)	15% (5)	17% (6)	18.8%(3)

Research decisions	4% (3)	9% (3)	14% (5)	12.5%(2)

Advocacy decisions	32% (24)	3% (1)	14% (5)	N/A

Other (write-in)	20% (15)^3^	15% (5)^4^	26% (9)^5^	31.3%(5)^6^

^1^respondents could indicate more than one role; ^2^respondents were asked to indicate one decision role only ^3^including, in order of frequency: education and training (of other professionals, or public awareness/outreach); other policy work; funding to implement public policy; ^4^management and mixed roles re: service, advocacy, research and planning; ^5^including project management, regulatory, policy recommendations, training, community presentations, policing, funding; ^6^including education/curriculum, funding, communication and knowledge translation.

To understand the relationships between the research team and stakeholders, we asked about their previous involvement with the VAW research program. In general, most respondents indicated low involvement, including receiving information on study findings through formal (Phase 1: 27%; Phase 2: 22%) and informal (32% and 25%, respectively) processes, or simply being 'aware of the VAW research program but not much else' (32% and 17%, respectively) (the decrease in this response over time may reflect respondents' exposure to earlier KTE efforts); 15% (Phase 1) and 22% (Phase 2) were 'not aware of the program until invited' to the event. Thus, the stakeholders to whom we spoke had varying, but generally not well developed, familiarity with the research program and its emerging findings.

### Knowledge uptake, sharing, use, and impact: Key findings

In order to examine key aspects of our KTE processes and the uptake and use of findings by stakeholders, the results of the study are presented across the study phases and according to the KTE activities and stakeholder reactions to them, while attempting to describe how the research knowledge was heard, shared, and used, and what, if any, early impact it may have had. Because the quantitative survey questions were highly complementary with the qualitative interview questions, we present related data together--that is, proportions of participants responding to survey questions are provided, and supporting quotes from write-in comments and interview transcripts are used to highlight and elucidate key findings regarding the KTE stages. Analysis of the content of the reactions, and their implications for VAW policy and practice, are beyond the scope of this paper.

### Knowledge dissemination and uptake

The focus of this section is to highlight participants' perceptions of our KTE processes, and identify which strategies were effective, and which were not.

Across both phases of this work, it was clear that participants placed significant value on the opportunity to attend in-person meetings with researchers, and with other stakeholders, and to think about and discuss research and its potential impact on decision-making in ways not usual to their daily work. Especially helpful, said participants, was the opportunity to meet and discuss the research with the researchers (80% of workshop and 92% of forum participants reported that they had the opportunity to meet and discuss the research with one or more of the VAW researchers and 97% and 100%, respectively, found this valuable), which helped them to assess the credibility and quality of the research. As one commented:

'It's hard to know the quality of [research] because we all skim these days. We all rely on just skimming through things and saying, 'Okay, is this something I should read in more depth?' And when you know the researchers by reputation, then I know that anything with their name on it is going to be worth reading.' [workshop follow-up interview, P06]

This ability to make a personal connection with a researcher enhanced awareness of the research and put it on their 'radar screen,' increasing the likelihood that future communiqués from the team would stand out. It was highlighted that face-to-face meetings are an important step in building relationships:

'...having the ability to [not just read] a paper but ... to hear from the researchers themselves and have the time and the luxury to digest and distil the information I think just keeps this research on top of the pile as opposed to getting lost in the shuffle of the many pieces of research that cross our desks.' [forum follow-up interview, P08]

In terms of the workshop and forum as information-sharing venues, there was certainly acknowledgement that these types of events increased their understanding of the complexity of the research process, including clarification of study findings and limitations; however, there was still a feeling from participants of wanting more--more clarity in what the data were saying, and more direction on what the data means for future practice:

'I think I was hoping to get more specific detail on some of the studies. More on identifying [and] responding to intimate partner violence in healthcare settings.' [forum follow-up interview, P05]

Participants also appreciated pre-circulated materials and handouts, and, especially at the forum, having the key messages 'well explained and clearly presented':

'...the research data had been boiled down to key messages and I know how difficult that was for the researchers. Really was much more impactful than a whole series of conclusions and you get lost in the information.' [forum follow-up interview, P03]

There was also some concern of information overload; however, this was balanced out with the appreciation for getting the larger picture, and making it relevant to a variety of stakeholders. These experiences were slightly different between the workshops, which presented much more detail regarding a series of individual projects, and the forum, which, based on feedback from the workshops, presented key messages concisely and clearly.

Another important experience for participants was the opportunity to provide feedback on the key messages (94% in the workshops and 98% in the forum reported this, and 98% of both groups found this valuable). In the workshop follow-up interviews, participants identified that providing feedback on a study still in progress was a novel and positive experience, especially for frontline staff from community-based services. As one workshop participant said:

'[The workshop used a] truly collaborative approach [with] respect for the input of the frontline. Often research is presented as a done deal, and frontline advocates, who I would say are the experts on the subject matter, are just treated as the consumers of the information versus the creators or holders of the information. I thought the [workshop] process was really respectful and that it worked really well.' [workshop follow-up interview, P07]

Several participants highlighted the necessity of a 'common language' and a common space for these sorts of discussions--and this type of forum was a good step in that direction, but that more still needed to be done.

When asked how responsive the project team was to their ideas and suggestions, most found us very or somewhat responsive (workshops: 80%; forum: 75%), while the rest indicated it was 'too soon to tell' or 'not applicable.' Individuals interviewed overwhelmingly described that the research team was genuine and respectfully listened to the different perspectives of VAW shared by participants.

Another significant benefit highlighted by participants was the opportunity to network with peers from across sectors and the multiple chances to engage in both individual and large and small group discussions: participants reported that they had an opportunity to meet other stakeholders (workshop: 94%; forum: 95%), which they found very valuable (99% in both samples); the opportunity to network over a meal was also appreciated. For some participants, the workshop provided a venue to share information about their organization and the services it offers.

A small number of workshop participants from the same group commented verbally to a member of the research team that those discussions were not well-facilitated, that the facilitation interfered with genuine discussion, or there was a single individual who dominated the conversation (and was not well-handled by the facilitator). Based on this feedback (which was provided informally and was not reflected in the written evaluations), formal facilitation was not used during the forum, and there were no expressed concerns regarding those discussions. Participants from both events liked the group format, especially mixing the stakeholders, as expressed by this person:

'... to be at a table with folks that were coming from different perspectives and having that conversation on how these messages were being interpreted by those different perspectives certainly gave me some food for thought in terms of how do you communicate these messages to people who really need to hear them. When you know they may [be] hearing different things than what you are trying to say.' [forum follow-up interview, P08]

When asked how valuable, overall, the events were for them and their work, the majority of participants indicated very or somewhat valuable (workshop 80%, forum: 89%); with the rest indicating it was 'too soon to tell' (workshop: 19%, forum: 11%). We also asked participants if they would like to stay connected with our KTE processes, and nearly all those who responded said they would (workshop: 97%; forum: 95%). While they indicated a range of preferences for ongoing communication with the research team, of note is the preference for electronic approaches, with over 75% preferring being sent electronic summaries of findings and/or links to the program website when new material is posted.

Despite the significant enthusiasm among workshop participants for the wiki-based 'online community of interest,' beyond an initial visit for its launch in March 2007 and completion of the follow-up survey, and despite encouraging reminders, the wiki was never used, and was eventually removed late in 2007.

### Knowledge sharing and use

This section presents data from the workshop and forum follow-up surveys and interviews specific to whether and how people shared what they had heard, and whether and how they had used the research findings. Table 4 provides an overview of the quantitative data from the two surveys, which is discussed below in light of what participants said during the interviews.

**Table 4:** Sharing and use of research results - follow-up surveys (Phases 1 and 2)

Question	Phase 1 Follow-Up Survey (3 to 6 months post-event) (n = 25, 8 missing)	Phase 2 Follow-Up Survey (6 to 8 months post-event) (n = 21)
Shared the research knowledge from the event?	YES = 88% (22) NO = 12% (3)	YES = 79% (15 of 19 who responded) NO = 21% (4)

**For those who responded YES**	**n = 22**	**n = 15**

Shared with (all that apply):		
Internal colleagues	42% (10)	100% (15)
External colleagues	48% (11)	47% (7)
Others	14% (3)	93% (14)

How shared (all that apply):		
Verbally	46% (11)	93% (14)
By email	4% (1)	13% (2)
Sent documents	8% (2)	47% (7)
Other^1^	25% (6)	53% (8)

Response to sharing		
Positively	43% (9)	27% (4)
Negatively	29% (6)	13% (2)
Mixed/Neutral	0% (0)	53% (8)
Can't tell/other	29% (6)	13% (6)

Used the research knowledge from the event?	YES = 40% (10) NO = 60% (15)	YES = 37% (7) NO = 63% (12) Missing = 2

**For those who responded YES**	**n = 10**	**n = 7**

How used (all that apply):	(1 missing)	(2 missing)
Conceptual	50% (5)	80% (4)
Symbolic	40% (4)	80% (4)
Instrumental	0	40% (2)

Have others used the research knowledge from the event?	YES = 12% (3) NO/Don't Know = 88% (22)	YES = 26% (5)^2^ NO/Don't Know = 74% (14) (2 missing)

^1^including: providing a link to the Research Program (n = 6), formal presentations or reports (n = 1) and informal discussions (n = 7); ^2^In the Forum follow-up survey we asked how others had used the knowledge, of the three responses, two indicated conceptual use, and one indicated instrumental use.

### Sharing research knowledge

As indicated in Table 4, in the three to six months following each event, the majority of participants indicated that they had shared the research with people in their organization and/or with external colleagues (workshop: 88%; forum: 79%); the information was shared verbally, electronically, through document-sharing and via reports and presentations:

'I did a lunch-and-learn with my colleagues about the research presented at this forum. I also presented the knowledge and my reflections on the event to our management team.' (forum follow-up survey, write-in).

Of interest is the type of sharing activity reported by those who attended the forum, all of whom reported more recipients of and approaches to sharing information, including internally, and also more broadly (93%) beyond 'colleagues.' Those who reported not sharing the information indicated that the primary reason for this was 'lack of opportunity.'

### Using research knowledge

The bottom part of Table 4 indicates that while there was some reported 'use' of the findings at the three- to six-month post-event point, this occurred much less often than the 'sharing' of knowledge, and was more consistent between the two phases, perhaps indicating that finding ways to actually integrate research evidence into decisions--especially after a relatively short period of time--is a much more complex process than simply 'passing it on.' In terms of use (and keeping in mind the small sub-samples who indicated use of any kind) across both phases (10 and 7 people, respectively), it was more common for the knowledge to be used symbolically and/or conceptually than instrumentally.

The follow-up interviews (at approximately 12 months post-event) helped shed some light on these processes. Reflections from participants indicated that in some cases the research findings increased their understanding (conceptual use) of issues related to VAW, and that when this was the case, findings were more likely to be used to reinforce or support current policies or programs within their organization (symbolic use). For example, in the 12-month forum follow-up interviews, ten participants used the information conceptually as background or context for other work they were doing. In this way, the information heard at the forum provided a new lens, and an opportunity to further consider their current practices:

'Well I think you take it more personally. I think you try to apply it to your everyday knowledge and your experience when you are front line.' [forum - small group 9]

Some participants used the research findings more instrumentally, for example incorporating it into in-house employee training, or into a report, or to update clinical protocols or guidelines. Forum participants who had used the information cited their attendance at this event as a major facilitator to using this knowledge.

However, we also learned that a number of participants would choose not to use evidence from the research program in their decision-making when it contradicted their personal experiences. These participants expressed discomfort with specific key messages (*e.g*., that screening is ineffective, or that pregnancy was not a risk indicator for current abuse). One workshop participant, even several years prior to completion of the screening trial, stated:

'From our experience we have already proven, or believe that we have proven that they [protocols for universal screening] have been incredibly effective and we will continue to have that policy and procedure in place ... So I would say it [the research evidence] has little or no impact ...' [workshop follow-up interview, P12]

And, during discussion of the actual trial results at the forum, another said:

'Well, we thought it would be unfortunate if the research was used to discredit the value of universal screening because intuitively we felt that universal screening made some sense even though the research doesn't show that it's probably worth the resources and the effort to do it. The benefits aren't worth that. But so we felt that perhaps it was premature to say that it wasn't.' [forum small group 5]

With regard to evidence around pregnancy and risk for violence:

'You know I've heard stories around this from women [that when they become pregnant, the abuse starts], so anecdotally I know that it's true. When the McMaster study said that pregnancy was not a [risk] indicator, I said, and was supported by other VAW people, 'That doesn't fit with our experience.'' [workshop follow-up interview, P16]

### Impact

It is well-acknowledged in the KTE literature that the most difficult thing to assess is the actual impact of new knowledge on specific policies or practices, or, ultimately, on health-related outcomes. We therefore examined the notion of 'impact' in terms of what our participants reported with respect to both the effect of them sharing the new knowledge with others, their own assessment of what happened when they used the research findings, and finally, the impact of the KTE processes themselves on respondents' decision-making.

### Impact of sharing--how do others respond to the knowledge?

The first aspect of 'impact' relates to how others reacted when participants shared the research findings with them. In general terms, we wanted to know whether the reactions were positive or negative (or neither), and what people might be planning to do with this new knowledge. Of the 22 respondents who shared the knowledge from the workshops, 43% indicated a positive reaction, 29% a negative reaction, and 24% were unsure of the reaction; write-in comments on the workshop follow-up survey, including one 'other' remark, indicated a range of reactions to the research, from colleagues preferring to wait for the final results (of the screening trial) to disappointment in hearing that abused women would prefer computer-based screening to speaking to a healthcare provider. When asked the same question, 27% of the 15 respondents from the forum indicated a positive reaction, 13% a negative reaction, and 53% a mixed reaction. Write-in comments, indicating the diversity of reactions, included:

'The findings somewhat discouraged some people, as they had seen screening as the answer to addressing this gap.'

'The screening issue continues to be a hotly debated subject and, while we are excited about the direction of the findings, those who are committed to screening continue to dispute the direction to expand beyond screening.'

### Impact of knowledge use--what happened?

We asked in slightly different ways in the two follow-up surveys the question of 'what happened' with regard to using the research findings. For the workshops, the focus was on the impact of use on a 5-point scale from very negative to very positive, or the option 'too soon to tell.' In that survey, of the 10 people who indicated they had used the research, six indicated the impact of this use was very positive or positive, one indicated it was negative, and three said it was too soon to tell.

In the forum follow-up survey and interviews, we asked 'what happened as a result of using the research findings on woman abuse screening?' and gave some specific response options, with respondents asked to check 'all that apply' and also comment on whether there was impact in their own work, and/or in the work of others. Only four people answered this question. Of those that did respond, the impact included actual or proposed/planned change to a policy, process, or course of action, and new points of discussion about these. None of these people expected 'nothing' to happen as a result of using the research knowledge, and when we asked participants to rank the impact the information has had on their work on a scale of 1 to 5 (5 is high), most (90%) felt quite positive about the impact, saying it had an impact of between 3 and 5. However, the difficulty in assessing 'impact' was reflected by this interviewee:

'Well that's a really hard question to answer because on the one hand absolutely no impact because we were already [decided against screening]...supporting that and they were supporting our work, so none. And then at the same time it's absolutely high because it affirmed in a kind of more objective way ... what we were doing. So externally I think it's a five [ranking]; internal for our own work, not so much.' [forum follow-up interview, P11]

In the forum follow-up survey, we asked if they planned to use the results in the future. Of the 18 who responded, 61% said yes and one person said no; the remaining six indicated it was too soon to tell. Write-in responses to the forum follow-up survey for this 'potential use' question included those who intended very specific uses: 'we plan to use the findings to develop formal woman abuse policy at our hospital as well as to direct provincial policy'; 'we are establishing a core public health program on the prevention of violence and abuse ... this issue will be discussed at our first working group meeting ...'; and the potential conceptual impact described by this participant:

'...may be useful in exploring why screening (or screening + brief intervention) should be seen as a prelude to treatment. I am interested in factors associated with treatment engagement and findings from this study may provide background support for the need to consider screening as a first-step in engaging people in treatment.' [forum follow-up survey, write-in]

In contrast, some were quite clear that the results contradicted their practices, and hence would not be used, or would be used selectively to support current approaches:

'the research indicated that universal screening for IPV does not cause harm; therefore, I will be using this research to continue to advocate for universal screening of IPV,' and: 'our students are currently taught to screen for abuse and this would create a mixed message.'

### Impact of KTE strategies and process

Finally, we wanted to understand the impact that participating in our KTE activities had on participants. When we asked whether they thought that being at one of our events would influence their decision making, among the workshop participants 42% said yes, 3% said no, and 49% said it was too soon to tell (four people either gave multiple responses or did not answer). These participants were also asked whether attending the workshop had influenced their decision making: 35% said 'yes' and 65% said 'no'; with regard to the overall impact that the workshop and related activities by the research program had on their work, the result showed modest impact (mean 2.65 on a 5-point scale from no to high impact). In the forum follow-up survey, we assessed the impact of our activities on participants slightly differently. In response to the question regarding whether the forum met their expectations on a number of domains, all but one respondent indicated that their expectations were met or exceeded regarding the kind of information presented (95%), the way the information would be presented (100%), and the usefulness of the information (100%). In response to the question regarding future attendance at a similar forum in the future, all but two respondents indicated that they would very likely or likely attend (88.9%).

### Suggestions for KTE processes

Participants also had many suggestions for both general and specific KTE strategies, while still being realistic around the complexity of the research:

'Those posters downstairs, it will be really great to have them shared, you know, even ledger size or legal size, if we could just take them back and hand them to people, because you know it's unfortunate that these large bodies of research have to be synthesized, cut down to sound bites, I know that's distortive of the message, really.' [forum small group 6]

Many reported that they found our efforts to interact and share knowledge to be helpful, innovative, and worthwhile, *e.g*., 'the effort to keep contact is noticeable and also valuable.' Participants acknowledged the difficulty in framing knowledge and communicating it to multiple types of stakeholders in one room, including selecting appropriate language (*i.e*., not too technical, but not disrespectfully simple), but also generally respecting different epistemological stances on what counts as 'evidence' and whose voices become privileged. This was very well-articulated by a number of participants, *e.g*.:

'... there's been a lot of taking up of academic language, evidence-based research ... show us an effective intervention, show us what we should be putting ultimately our energy and funding behind. But that's really difficult to do in some sectors, [VAW] specifically, that emerged really around kitchen tables and grass roots ... so how do you reconcile the need for evidence-based research to propel forward program development, policy development in the area versus the kind of community-based knowledge and expertise that really gave way to the emergence of these programs and services in the first place?' [forum small group 6]

## Discussion

This paper has described a series of KTE activities conducted by a specific research program as it progressed across time. This longitudinal, mixed methods approach to evaluating the KTE process and its outcomes is rare in the literature, and, to our knowledge, has not been reported in violence research. Below, we reflect on several of our main findings in the context of existing literature so as to comment on whether what we found is consistent or inconsistent with what was previously known, and what new findings might advance our thinking in this area.

### Talk, trust, and time

A key finding that emerged from our data was the importance of what we call the 'three Ts'--talk, trust, and time. Repeatedly, our participants cited our willingness to actively engage with them, in person, as crucial to developing both credibility, and, over time, trust in the research team and its products. While the importance of personal relationships to facilitate KTE processes is not a new finding [21], and more recent work highlights the fact that highly interactive relationships significantly facilitate the adoption of tailored KTE innovations [34], our longitudinal approach allowed us to begin to examine how these processes unfold. Participants appreciated our efforts to present findings in ways most useful to them, acknowledging that this is not easy for researchers trained in very specific forms of research reporting. Similarly, and consistent with the findings of others [35,36], there was an appreciation of our willingness to engage stakeholders respectfully, which was expressed most strongly by those who identified with 'the frontline.'

One interesting finding was that this emerging trust, or, at least, willingness to listen and engage, was not universally felt by all participants--a number of stakeholders actively resisted some of the messages that they found problematic, either because they contradicted long-held beliefs, or they did not fit with their own direct experiences. This resistance was most often expressed through critiques of the research methodology, especially issues of sample limitations (*e.g*., that all women in the study spoke English and could self-complete the research forms, or that research is more structured and resourced than 'real' clinical practice) and the notion that this is 'only one study'--both of which are valid concerns that were considered by the research team in framing the findings and included in presentation of key messages. However, when set in the context of a significant body of work including multiple systematic reviews examining the healthcare response to VAW [11,13,14], our conclusions are consistent with a broader consensus that there is a lack of proven benefit for universal screening--at least from an 'evidence-based' perspective [37]. Analysis of these 'resistance discourses' is ongoing, but the fact that evidence often bumps up against values and beliefs must be taken into account not only when designing KTE strategies, but also in determining realistic expectations for their success. As stated by Jacobson *et al*., 'knowledge utilization is facilitated when there is congruence between the implications of the research and the particular ideology that dominates the user group context' [21]. We certainly found this to be the case.

### Assessing knowledge use

In terms of how participants reported using the research findings, a few interesting observations can be made (acknowledging small sub-samples). First, following the 2006 workshops, while some participants did report using the findings, all indicated that this was at the conceptual or symbolic level, which is not surprising given the type of information presented--descriptive/epidemiological studies and an RCT that was more about process than outcome. After the 2009 forum, where we presented results and recommendations regarding screening, we heard more (though still few) examples of instrumental use, along with conceptual and symbolic use. Use was specifically related to both the types of decisions being made, and to where people were in the decision process: for those actively making decisions, specific instrumental applications were described; for those planning to make or requiring support for previous decisions, use was at a more conceptual or symbolic level-- *i.e*., to 'justify' or 'convince.' Consistent with previous literature, however [24], it is not surprising that instances of reported instrumental use were infrequent, especially among these kinds of stakeholders [18].

Of note, some people reported using the results to support current beliefs and practices regardless of whether the research findings actually matched these. While we do not have the space within this paper to fully explore this, initial analysis indicates that this had as much to do with the nature of the results (not completely consistent across all outcomes), as with the nature of the content area. Further exploration of how and why people make specific interpretations of research evidence, and the roles of such things as individual cognitive processing [38], especially cognitive dissonance reduction, are warranted to fully understand the interplay between knowledge uptake, interpretation, and use.

One issue that became clear to us during our KTE events, as well as a number of other presentations and discussions of the screening trial findings, is the serious concern about unintended consequences of knowledge uptake and use. Some participants were very worried that those already resistant to 'doing something' about VAW, for example clinicians hesitant to ask about exposure to violence and open a clinical 'Pandora's box' [39], would use our screening trial results to support their non-action. Ethical application of KTE strategies, and a full examination of potential unintended, as well as intended, consequences of knowledge sharing, must be considered during the KTE planning stages. However, it is not only difficult to anticipate outcomes that are unintended, but also to determine the balance of benefits and harms of sharing or not sharing important new research evidence. As has been argued: '[t]he sense of urgency to translate for public greater good and system improvements should be tempered with clear messages that translation is an ethically-bound process that should be judiciously appraised' [40].

We would therefore argue that thinking about the use, non-use, and impact of research knowledge as staged, highly-specific processes is more nuanced than attempting to determine whether specific policies or practices actually change to incorporate new evidence. For example, our findings that some stakeholders would not change their views if the evidence was felt to challenge their individual and established beliefs would require, if we were to determine 'effective' KTE strategies in this situation, us to follow both 'adopters' and 'resisters' across time. This could determine, what, if anything, might influence them to change their attitudes or practices in response to research evidence. As has been noted by others grappling with the issue of 'user context,' these issues are complex [22].

### Limitations and lessons learned

The data presented in this study are entirely descriptive, and small sub-samples preclude statistical analysis or definitive statements on such issues as actual 'use' and 'impact,' or type of use by stakeholder setting or role. However, the consistency of our key findings across time, and the triangulation of qualitative findings with quantitative data, lends credibility to our results. Many of our findings are also consistent with existing KTE literature, for example with regards to stakeholder preferences, barriers, and facilitators, including timing of decisions, resource implications, competing demands, and diverse perspectives [17,18,23-25,35]. However, one thing that this study adds is data regarding a process we might frame more as 'mobilization of ideas,' rather than 'dissemination,' 'implementation,' or even 'knowledge translation.' Our decision to communicate concurrently with multiple types of stakeholders, including policy makers, healthcare practitioners, community service providers, and women's advocates, was made *a priori *and in the context of this specific research area. As those of us leading the research team learned from earlier stages, the potential risks of seeming to privilege communication with one group over another, even in terms of who is contacted first, were a concern, and we adopted the principle of speaking with all stakeholders at once as one that was both egalitarian and, more importantly, respectful. This approach presented its own challenges, particularly in terms of selecting appropriate message formats and channels, and ensuring that recommendations were applicable across sectors. This may have 'diluted' the potential impact of messages, because they were not as tailored as they might otherwise have been. That said, our results lend support to the focus on developing new ways of thinking about, and new strategies for, community-oriented KTE, especially in multi-stakeholder contexts, an area emerging in recent KTE literature [41,42].

At a more practical level, a challenge to staging our activities was getting people, given busy schedules and competing demands, to attend events (*e.g*., over 400 invitations were sent for the events, 168 attended them).

Another interesting issue is the use of a KTE activity at what was essentially a partway point through the overall research program, which had as its ultimate goal addressing the effectiveness of screening women in healthcare settings. Given the number of precursor studies required to develop the knowledge needed for the ultimate screening trial, we felt, in 2006, that we had a significant amount of important data that would inform key knowledge gaps. It was also thought important to share our progress, and to engage stakeholders who might be wondering where the project stood. However, one thing that became clear during the workshops was that people were expecting to hear results of the screening trial, and when these were not available, some were frustrated. Thus we were left to balance the utility of these 'research in progress' events as both relationship and trust-building, and/or trust-diluting with the potential to disappoint. Our analysis indicates that more participants appreciated the opportunity to hear about and provide input while the research was still in development, than did those who voiced concern over premature engagement.

In terms of KTE strategies that clearly did not work as anticipated, the experience with the online, wiki-based 'community of interest' stands out. However, while disappointing in terms of the return on investment, this was an interesting finding, and consistent with recent research [34,43] indicating that even highly tailored, integrated KTE innovations may not be adopted, even if stakeholders express a desire for them. For example, Driedger *et al*. [34] describe the relatively low uptake of a novel geographic information system (GIS)-based mapping system in a community setting, finding that the strongest facilitator of knowledge use was the close personal working relationships between the data analyst and the knowledge user.

Finally, perhaps the clearest 'lesson learned' from this process was negotiating the space between 'too much' and 'not enough' research detail in communicating with our diverse audiences. It is a significant departure for researchers to 'boil down' results to two or three high-level messages, without the usual justifying data, statistics, and qualifiers. Developing messages and KTE strategies that allowed us to communicate with all stakeholders at once and not privilege one group over another, while being 'evidence-based' in our messages, was perhaps the most challenging aspect of this project, and one that could not have been contemplated if we had relied solely on one-off, one-way transmission of findings using static, non-interactive approaches.

### Future research

Future research to assess knowledge use, using longer follow-up intervals to address the need to allow time for use to occur, is needed. This will require using methods and measures that can document and assess the impact of other information received by users (*i.e*., 'contamination') after or concurrent with the KTE activities being evaluated, as well as threats to 'message fidelity' (recall bias, conflicting messages, *et al*.) across time.

Also required is a more thorough exploration and analysis of how the concept of 'knowledge sharing' fits, or not, in currently used knowledge utilization frameworks. Emerging evidence in related areas--including how communities of practice facilitate knowledge use [42], and knowledge/evidence as a form of social capital [44] in organizational social networks [45]--points to the need for this kind of analysis. Perhaps the kinds of knowledge-sharing strategies used in our work are best suited for enabling knowledge brokering, where individuals hear new research and bring it back to their organizations/colleagues, where it may, immediately or ultimately, influence decisions. Developing ways to assess these complex processes, including the role of relationship development and 'trust' between researchers and research-users, and mechanisms to understand the effects of processes such as cognitive dissonance that may influence message uptake, is a key next step in this kind of knowledge translation work [41].

In summary, we found that a number of factors influence the uptake, sharing, and use of new research knowledge related to identification, in healthcare settings, of women exposed to violence. These factors, as outlined in Oh and Rich [17], are specific to the information itself, characteristics of those receiving the messages and their knowledge use contexts, and the nature of the knowledge gap being addressed. In particular, the factors that stood out, as reported by participants, included the potential concordance or discordance between the kind of (research-based) evidence that our studies provided, and other kinds of knowledge, including practice-based experiences, in determining knowledge uptake, sharing, and use. Related to this, the nature of the research area--where beliefs are often strongly held--makes KTE a particular challenge. Perhaps hindering the overall process was the potential for some of our results to be viewed as ambiguous or inconclusive, which may have presented the opportunity for multiple interpretations and applications of the 'bottom line.' Finally, while there was some indication that specific stakeholder setting (*e.g*., organizational versus individual practitioner; policy versus clinical) influenced the potential use and impact of the findings, sub-samples were too small to explore this more fully.

## Conclusions

KTE in multi-stakeholder contexts is complex and resource-intensive, and must acknowledge and respect the values of stakeholders while balancing the objectivity and neutrality of the research and researchers. One-size-fits-all approaches to KTE do not address the complexities and particularities of specific contexts [46], nor the interaction of contextual factors with 'evidence.' Stakeholders are likely to use a much-broader definition of 'evidence' than researchers when interpreting new research knowledge, and their acceptance, uptake, and use of the new knowledge will in part depend on how it meshes with their own beliefs, values, 'professional craft knowledge,' [22] and experiences, and how it might 'fit' with their decision-making context. 'Context' in this respect includes such things as organizational culture, but also how the actors in the context are 'situated' to the new knowledge, which includes many very specific factors, such as previous actions or policies, as well as the timing of decisions that may (or may not) incorporate research findings. Our findings in this area have implications for how we think about 'knowledge translation' more broadly. In fact, in an area such as violence against women, the 'evidence-based medicine' framework may well be inappropriate for knowledge designed to inform not only health services, but also broader community and social services, and to enlighten women and their advocates. As Davies *et al*., using a social research frame, articulate ''knowledge interaction' might more appropriately describe the messy engagement of multiple players with diverse sources of knowledge, and ... 'knowledge intermediation' might begin to articulate some of the managed processes by which knowledge interaction can be promoted' [47]. New theories and methodologies that can assess and explain 'knowledge mobilization' as a construct related to, but distinct from, current 'knowledge translation' approaches are required.

## Competing interests

The authors declare that they have no competing interests.

## Authors' contributions

CNW prepared the outline and drafted all sections. SMJ and SLS contributed specific sections of the results. CNW, SMJ, and HLM obtained funding for both phases of the study. All authors participated in data collection and/or staging of KTE events, reviewed all sections and provided input on interpretation of results. All authors read and approved the final manuscript.

## Supplementary Material

Additional file 1 **Key Message Development for the VAW Research Program and Messages Presented at January 2009 Family Violence Knowledge Exchange Forum and Additional Background Information About the McMaster VAW Research Program**. Click here for file

Additional file 2 **Additional details regarding study processes, data collection tools, methods and samples**. Click here for file
