# Supplementary material for: Talk, trust and time: a longitudinal study evaluating knowledge translation and exchange processes for research on violence against women
Source: Implement Sci. 2011 Sep 6;6:102. doi: 10.1186/1748-5908-6-102 (PMC3178499; doi:10.1186/1748-5908-6-102)
Supplement: Additional file 1 — Key Message Development for the VAW Research Program and Messages Presented at January 2009 Family Violence Knowledge Exchange Forum and Additional Background Information About the McMaster VAW Research Program. [file 1748-5908-6-102-S1.DOC]

Wathen et al. *Talk, Trust and Time: A Longitudinal Study Evaluating Knowledge Translation and Exchange Processes for Research on Violence Against Women*

**Additional file 1:**

Title: Key Message Development for the VAW Research Program and Messages Presented at January 2009 Family Violence Knowledge Exchange Forum and Additional Background Information About the McMaster VAW Research Program

Process:

The process for key message development in both phases of the KTE project was structured and iterative, including: solicitation, using feedback forms, of potential messages from members of the research team, including knowledge user partners; collation of these results; then a process of discussion and synthesis with our policy partners/funders regarding specific messaging and format. In Phase 1, the result was a project-by-project set of messages, presented in significant detail at the 2006 Workshops. In Phase 2, in an attempt to further distil, refine and synthesize messages across all completed studies, the process also included a policy briefing in December 2008 between the core research leads and a small group (n = 8) of policy personnel from three Ontario government Ministries (Health, Community and Social Services and Ontario Women’s Directorate), previously involved in either funding or having design input into the research. At the briefing, researchers presented the results and discussed potential key messages. In early January 2009, the research team presented the trial results at a larger briefing that included policy analysts, managers, and directors with VAW portfolios from across multiple Ministries. Feedback from these two meetings helped refine the Key Messages presented at the 2009 Forum.

Additional details and copies of the tools used in these processes are available from the corresponding author.

Messages Presented at the 2009 Forum

(presented in 6 slides)

*1. IPV Screening Effectiveness Trial*

- 18-month randomized controlled trial (N = 411) of screening women in 26 Ontario health care settings for past-year exposure to IPV.
- Screening may have modest benefits for life quality and some, but not all, aspects of abused women’s mental health, including depression. These may not be clinically important changes.
- Screening results in a small non-significant reduction in exposure to violence when compared to no screening.
- Our screening process over-identified women as experiencing abuse and many women were screened to identify one who disclosed past-year IPV.
- 44% of screened women discussed IPV with their clinician compared to 7.5% of non-screened women.
- Screened and control group women had no differences in the frequency of using violence-related health and social services.

*2. Educating HCPs to identify and respond to abuse*

- Large-scale studies in Ontario health care settings have clearly identified red flags for increased risk of IPV, e.g. Unemployment or alcohol or drug use in male partners, depression and somatic symptoms in women.
- These provide clinical indications for health care providers (HCP) to ask women about abuse, especially when more than one factor is present.
- However, large surveys of HCPs, university and college programs and professional organizations indicate that health and social service providers in Ontario are not well-prepared to recognize indicators of abuse, or to ask women about such exposures.

*3. Public awareness & services*

- Based on surveys and focus groups, members of the Ontario public underestimate the prevalence of IPV and its consequences for women and families.
- Interventions proven effective for reducing violence and/or improving life quality in abused women identified in health care settings are lacking.
- While services for abused women exist in many (but not all) Ontario communities, women may not be accessing them, even when provided with information about resources when they disclose abuse.
- Rigorous evaluation of what is effective in reducing violence and improving health should be a key priority.

**BOTTOM LINE**

- Screening – had modest benefits on some outcomes, and did not cause harm, but clinical impact and feasibility are concerns

Considerations:

- Case finding – evidence for specific clinical indicators to prompt asking about abuse – PRIORITY
- Professional education – sorely lacking – PRIORITY
- Services – lack of proven effective intervention linked to health-care; evaluation required – PRIORITY
- Public perceptions – still lagging

List of McMaster VAW Research Program Projects and Selected Related Publications

*Projects*

1. Women’s Views, Experiences & Needs for Woman Abuse Screening
2. Health Care Providers’ Knowledge, Attitudes and Practices to Woman Abuse Screening
3. Patterns of Screening for Woman Abuse in Public Health Practice
4. Population Attitudes Towards Screening
5. Meta-Analysis of Risk Correlates for Woman Abuse
6. Development of the Risk Indicator Tool (RIT) for Woman Abuse
7. Public Health Nurses’ Roles in Asking About and Responding to Intimate Partner Violence (IPV) in Home Visitation
8. Development of the Consequences of Screening Tool: A multidimensional assessment of potential harms from screening for interpersonal violence in health care settings.
9. Women’s Disclosure of Intimate Partner Violence to Health Care Providers in Urban Emergency Department Settings: A Grounded Theory Study
10. Randomized Trial of Screening Tools and Approaches
11. Randomized Controlled Trial of Universal Screening for Woman Abuse
12. How Information Helps Abused Women: A Prospective Cohort Study in Health Care Settings
13. Cost-Effectiveness Evaluation of Screening
14. Evaluation of Feasibility & Acceptability of Screening to Women and Health Providers

(plus specific secondary and sub-analyses)

*Selected related publications*

MacMillan HL, Wathen CN, Jamieson E, Boyle MH, Shannon HS, Ford-Gilboe M, Worster A, Lent B, Coben JH, Campbell JC, McNutt LA; McMaster Violence Against Women Research Group. Screening for intimate partner violence in health care settings: a randomized trial. *JAMA*. 2009 Aug 5;302(5):493-501.

Wathen CN, Tanaka M, Catallo C, Lebner AC, Friedman MK, Hanson MD, Freeman C, Jack SM, Jamieson E, MacMillan HL; McMaster IPV Education Research Team. Are clinicians being prepared to care for abused women? A survey of health professional education in Ontario, Canada. *BMC Med Educ*. 2009 Jun 18;9:34.

Wathen, C.N., Jamieson, E., MacMillan, H.L. Who is identified by screening for intimate partner violence? *Women’s Health Issues* 2008;18(6):423-32.

Jack, S.M., Jamieson, E., Wathen, C.N., MacMillan, H.L. The feasibility of screening for intimate partner violence in postpartum nurse home visits. *Can J Nursing Research*, 2008;40(2): 150-170.

Wathen, C.N., MacMillan, H.L. Self-report, medical staff interview, and physician interview had similar effectiveness for screening for domestic violence in women. *Evidence-Based Nursing*, 2008;11(2): 45.

MacMillan, H.L., Wathen, C.N. American Medical Association Symposium: Identification of Intimate Partner Violence in Health Care Settings: What’s the Evidence? *DePaul Journal of Health Care Law* 2007;11(1):69-87.

Wathen, C.N., MacMillan, H.L., Lent, B., Jamieson, E.. Whether, when and how to ask about intimate partner violence**. *Ann Fam Med*** 2007 **October 31.** Available: <http://www.annfammed.org/cgi/eletters/5/5/430>

Wathen, C.N., Jamieson, E., Wilson, M., Daly, M., Worster, A., MacMillan, H.L. Risk indicators to identify intimate partner violence in the emergency department. *Open Med* 2007; 1(2):e113-22. Available: <http://www.openmedicine.ca/article/view/63/62>

Gutmanis, I., Beynon, C., Tutty, L., Wathen, C.N., MacMillan, H.L. Factors influencing identification of and response to intimate partner violence: a survey of physicians and nurses. *BMC Public Health* 2007; 7:12 [doi:10.1186/1471-2458-7-12.]Available online: <http://www.biomedcentral.com/content/pdf/1471-2458-7-12.pdf>

Wathen, C.N., MacMillan, H.L., Jamieson, E. Screening for intimate partner violence [Letter]. *Am J Prev Med* 2006;31(5):453.

MacMillan HL, Wathen CN, Jamieson, E, McNutt, LA, Worster, A, Lent, B, Webb, MA for the McMaster Violence Against Women Research Group. Randomized trial of approaches to screening for intimate partner violence in health care settings. *JAMA* 2006;296(5), 530-536.

MacMillan HL, Wathen CN. Family violence research: Lessons learned and where from here? *JAMA* 2005;294(5): 618-620.

McClennan SK, Worster A, MacMillan HL. Survey of domestic violence screening protocols in Canadian emergency departments. *Can J Emerg Med* 2005;7(3):185.

Taket A, Wathen CN, MacMillan HL. Should health professionals screen all women for domestic violence? *PLoS Medicine* 2004;1(1):7-10 (e4-e9 online).
