# Supplementary material for: Talk, trust and time: a longitudinal study evaluating knowledge translation and exchange processes for research on violence against women
Source: Implement Sci. 2011 Sep 6;6:102. doi: 10.1186/1748-5908-6-102 (PMC3178499; doi:10.1186/1748-5908-6-102)
Supplement: Additional file 2 — Additional details regarding study processes, data collection tools, methods and samples. [file 1748-5908-6-102-S2.DOC]

Wathen et al. *Talk, Trust and Time: A Longitudinal Study Evaluating Knowledge Translation and Exchange Processes for Research on Violence Against Women*

**Additional File 2:**

Title: Additional details regarding study processes, data collection tools, methods and samples.

Specific questions used in Phase 2 (Forum) Small Group Discussions

*1) What do these messages mean to you? What resonated with you and what did not? Why?*

*2) How do these messages fit or not fit with current policies, practices and/or values?*

*3) What should happen with these messages? How will/could they be used to inform practice and policy?*

*4) What challenges will people face to integrate these findings into practice and policy decision making? How do we overcome these?*

Details of the participating samples, by Phase and data collection method.

*Phase 1 Workshop evaluation and follow-up survey and interviews* - In total, 75 of the 82 (91.5%) workshop participants completed the evaluation survey. Of these 75 respondents, 20 were purposefully selected and interviewed one to three months following the workshop. Approximately 40 stakeholders joined the online community of interest in March 2007, and 33 of these completed the follow-up survey.

*Phase 2 Forum evaluation, small groups, follow-up survey and interviews* – During the Forum, participants formed 10 small groups whose discussions were the basis of the analysis of immediate impact of the research. Of the 87 attendees, 76 were external to the McMaster VAW Research Program Team, 38 of these completed the post-workshop evaluation survey (50% response rate) and 26 of these gave their permission to be contacted for follow-up. Of these, 21 (81%) completed the online follow-up survey approximately 6 months after the event, and 12 were purposefully selected for interview 6-12 months after the Forum.

Other KTE activities

In August 2009, the screening trial was published in the *Journal of the American Medical Association* [16]. This necessitated preparation by the lead authors of a press release, and ‘talking points’ in the event of media interest. Following the journal’s embargo period, the lead authors conducted several media interviews locally and for the Canadian media, as well as a podcasted ‘clinical conversation’ for a US medical website (<http://podcasts.jwatch.org/?p=573>). We therefore included questions about media exposure in relevant follow-up surveys and interviews.
